# The Meaning of Sense of Coherence (SOC) in Persons with Late Effects of Polio—A Qualitative Study

**DOI:** 10.3390/ijerph19106314

**Published:** 2022-05-23

**Authors:** Maria Nolvi, Anna Forsberg, Christina Brogårdh, Lars Jacobsson, Jan Lexell

**Affiliations:** 1 Department of Health Sciences, Lund University, S-221 00 Lund, Sweden; anna.forsberg@med.lu.se (A.F.); christina.brogardh@med.lu.se (C.B.); lars.jacobsson@med.lu.se (L.J.); jan.lexell@med.lu.se (J.L.); 2 Department of Neurology, Rehabilitation Medicine, Memory Disorders and Geriatrics, Skåne University Hospital, S-221 85 Lund, Sweden; 3 Department of Thoracic Surgery, Skåne University Hospital, S-221 85 Lund, Sweden; 4 Department of Rehabilitation Medicine, Sunderby Hospital, S-971 80 Luleå, Sweden

**Keywords:** adaptation, psychological, disabled persons, post-poliomyelitis syndrome, qualitative research, rehabilitation, self-management, sense of coherence

## Abstract

Sense of Coherence (SOC), comprising comprehensibility, manageability and meaningfulness, is important for the sense of living a good life with Late Effects of Polio (LEoP). However, there is a lack of knowledge about the meaning of these three components. The aim of this study was to explore in-depth the meaning of SOC among persons living with LEoP, in terms of comprehensibility, manageability and meaningfulness. A directed content analysis was performed based on individual interviews with 7 men and 7 women with LEoP (mean age 73 years). SOC in persons with LEoP existed in two overarching themes that were closely intertwined: a state of motion and a state of being. The state of motion comprised active approaches, choices and actions, and was a process aimed at achieving a stronger comprehensibility, manageability and meaningfulness. The state of being comprised the comprehensibility, manageability and meaningfulness that the persons currently experienced. A profound understanding of SOC as both a state of motion and state being is essential for rehabilitation professionals when providing self-management support to persons living with LEoP. This understanding can increase their sense of living a good life and also be used in the rehabilitation of other life-long conditions.

## 1. Introduction

Many persons who experienced paralytic polio in their childhood have adapted successfully to a life with a remaining disability. However, decades after their initial polio infection, they may develop new symptoms known as Late Effects of Polio (LEoP) or Post-polio syndrome [1]. Common symptoms among persons with LEoP are muscle weakness, general fatigue, pain during activities and at rest, cold intolerance and breathing difficulties [1]. This can affect their sense of control, their mobility and their manageability of meaningful activities in daily life [2], which may have a psychological impact and lead to a feeling of being on a downward slope without control [3]. Despite a gradual functional decline, studies have shown that persons with LEoP are generally satisfied with their lives [4,5]. Thus, many persons with LEoP have adapted successfully also to their new disability and continued to live good lives. In order to continuously support persons with LEoP, rehabilitation professionals need a deep and broad understanding of factors underlying this sense of living a good life.

One factor that is important for a sense of living a good life is “salutogenesis”, introduced by Aaron Antonovsky [6]. Salutogenesis, meaning the origin of health, is focusing on factors promoting health and well-being, instead of focusing on illness and disease. Antonovsky found that the necessary elements in salutogenesis are “comprehensibility”, “manageability” and “meaningfulness”, which together make up the sense of coherence (SOC). Accordingly, persons with a strong SOC can understand and handle challenges, such as the stressors caused by a disability, and are motivated to deal with them. Among other factors, SOC has been related to mental health [7,8] and successful adaptation to a disability [9]. In persons with LEoP, we have shown that SOC is generally similar to that of the non-disabled population [10] and that SOC is more strongly related to life satisfaction and perceptions of self than the actual LEoP-related disability [11]. Recently, we also demonstrated that persons with LEoP who have a strong SOC avoid using maladaptive coping behaviors that might worsen the situation [12]. In summary, our findings suggest that persons with LEoP and a strong SOC can, despite a new disability, handle its inherent challenges and continue to live good lives.

Thus, SOC seems to be a key factor for persons with LEoP, their successful management of the stressors related to growing old with a disability and their sense of living a good life. However, there is a lack of knowledge about the in-depth meaning of comprehensibility, manageability and meaningfulness, the three components of SOC, expressed by persons living with LEoP. A deeper understanding of salutogenesis might be essential for targeted self-management support and possibly increasing the sense of living a good life. Thus, the aim of this study was to explore in-depth the meaning of SOC among persons living with LEoP in terms of comprehensibility, manageability and meaningfulness.

## 2. Materials and Methods

### 2.1. Research Design

This study has a qualitative research design and stems from the assumption that there are multiple subjective realities. Thus, we have adopted an interpretivist approach based on the participants’ narratives guided by the SOC framework. Individual interviews and a directed content analysis [13] were used to explore the meaning of SOC in persons with LEoP. This design was chosen as we aimed to obtain a deeper and broader understanding of an existing framework—SOC—and add further knowledge to this concept.

### 2.2. Participants

The participants were recruited from a clinical database including persons with LEoP who had undergone interdisciplinary rehabilitation at a University Hospital in southern Sweden. They had all taken part in our previous studies of SOC and associated factors in persons with LEoP [10,11,12]. Inclusion criteria were verified LEoP and living in their own homes. Exclusion criteria were being unable to take part in an interview and having a clinically verified depression. The participants were selected to represent different gender, age, marital status and educational background. We aimed to achieve an equal gender distribution, an age range that covers most persons with LEoP, participants that were both single and married/cohabiting, had a different educational background, and had long-term experience of LEoP, were cognitively intact and not depressed, and thereby could provide rich narratives.

In total, 21 potential participants were contacted by letter. Five persons did not respond to the invitation. Among the 16 persons who volunteered to participate, two were excluded; one due to a hearing loss and clinical depression and the other as a result of a very late response when the other interviews had already been completed. Consequently, we included 7 men and 7 women with a mean age of 73 years (SD 5). All 14 participants had a confirmed history of acute poliomyelitis in their childhood and were experiencing new symptoms after a period of stability that had lasted for at least 15 years.

As part of the verification of previous polio, they had undergone an electromyogram (EMG) in all four limbs, and all participants had EMG findings indicative of previous polio in at least one limb. All participants lived in their own homes and at the time of the interview none were considered to have clinical depression or any major cognitive or physical impairment that would impede their participation.

Data on the 14 participants are presented in Table 1. Men and women were equally represented and the age range of 65–84 years covers most persons with LEoP, at least in the western world. Their disability level also varied; some persons were quite ambulatory, whereas others used a wheelchair as their main mode of transportation. Both married/cohabiting and single persons were included, providing a rich source of experiences.

### 2.3. Procedure

Before inclusion, the participants received a letter with information about the study and gave their written informed consent, which they sent back in a prepaid envelope. They were informed that data could not be traced to any of them, and that they had the possibility to withdraw at any time and that this would not affect their right to receive rehabilitation or healthcare at a later stage. The first author, who also performed all interviews, contacted the participants by phone and arranged a time and a place for the interviews between May and July 2021. Eight interviews were performed face-to-face and the remaining six digitally. The face-to-face meetings were conducted in the participants’ homes (n = 7) or at the office of the research team (n = 1). All interviews were performed individually, except for one where the participant’s spouse was present during the interview but did not become involved.

### 2.4. Data Collection

Data were collected through semi-structured interviews. An interview guide was developed based on the SOC categories, i.e., comprehensibility, manageability and meaningfulness. The interview guide was pilot tested on the first three participants, which resulted in minor changes. The questions explored the meaning of SOC among the participants, such as: “Could you please tell me what you understand about post-polio and why you have your present symptoms?”; “Could you please tell me how you manage your post-polio?” and “What gives you meaning in your everyday life?”. Follow-up questions were posed, such as “Could you please describe what you mean by…” and “Could you give examples…”. The interviews lasted between 10 and 56 min (average 31 min). All interviews were recorded using a digital recorder, transcribed verbatim and the accuracy of the transcripts was checked against the audio files. After the 14th interview, we were convinced that we understood what we saw, could identify the relevant forms of the results, and that it appeared consistent. Thus, we decided that we had reached saturation and terminated the data collection.

### 2.5. Data Analysis

The directed content analysis [13] included a five-step procedure:

1. Achieving an overall impression of the content by reading all the interviews and obtaining a naïve understanding.
2. Text units involving comprehensibility, manageability or meaningfulness were identified and copied into an individual matrix sheet for each of the SOC categories.
3. In the matrix sheet, each text unit was analyzed and interpreted, after which the meaning units were condensed into codes.
4. Codes were sorted into subcategories, covering the meaning of comprehensibility, manageability and meaningfulness.
5. Identification of the two overarching themes.

The analysis and interpretation were mainly performed by the first author (MN) together with the second author (AF), who has long experience in qualitative research, and further validated by the third author (CB). All authors had access to the transcripts of the full interviews and the findings were discussed among all authors throughout the process.

## 3. Results

The analysis revealed two overarching themes that were closely intertwined: SOC among persons with LEoP existed in a ***state of motion*** and in a ***state of being***, visible in all three categories of SOC. The state of motion comprised active approaches, choices and actions, and was a process aimed at reaching stronger comprehensibility, manageability and meaningfulness. The state of being comprised the comprehensibility, manageability and meaningfulness that the persons currently experienced. SOC became profound when the person could feel certainty, act according to his or her own free will and enjoy participation and meaningful social interactions. The meaning of each main category of SOC is presented in subcategories indicated by bold italics in the text and summarized in Table 2. To add transparency and trustworthiness to our findings, quotations from the participants are also provided.

### 3.1. The Meaning of Comprehensibility

The motion in comprehensibility occurred as a learning process of sense-making, i.e., understanding cause and effect, and how and why one’s health condition was affected. The process of sense-making included various learning strategies. ***Simplifying by using metaphors and making things concrete*** meant breaking down difficult information into pieces that were easier to grasp, e.g., that the nervous system works like an electric system.

“*It’s about the electrical system that drives us. When I got polio, part of my spinal cord was shut down and all the electronic wires were out of current. They didn’t work anymore. But they (rehabilitation professionals) told me that the body is remarkable as it makes different nerve cells replace the deficient ones. That is why some symptoms disappear after a while. However, because some parts of the nervous system become overloaded and lose their function you get post-polio, if I understand it correctly.*”

(P1)

Another learning strategy was ***re-evaluating information and identifying patterns***. The participants considered some information they had received as young persons with polio no longer valid. A few had been advised not to participate in physical activities at school in contrast to newer advice about being active at an appropriate level. In addition, knowing that the condition is progressive and that their functioning could deteriorate further in the future meant that they continuously needed to re-evaluate the “appropriate level of activity”. The participants experienced fatigue and pain, and identified patterns between these experiences and excessive activities, strained muscles and overweight. However, it was often difficult to distinguish whether the problems were due to LEoP or aging itself.

“*Walking is getting harder and harder. I get far more tired and it takes longer to recover. I guess that’s how I noticed it predominantly…. But it´s hard to distinguish if it´s due to old age or post-polio.*”

(P3)

The strategies of simplifying, re-evaluating information and identifying patterns led to ***developing personal models of explanation of illness***. Based on the information they had and the patterns they could identify, they gained an understanding of their current condition.

“*Since I´ve got these arrangements (orthoses), I´ve noticed that some tiny muscles don´t need to work as before, which might have made them wither and led to an even more reduced muscularity in the leg, as I haven´t had to use these muscles.*”

(P3)

Comprehensibility as a state of being meant understanding the demands of being a person with LEoP. This was characterized by trusting rehabilitation professionals and ***following*** their ***instructions***. Rehabilitation professionals’ instructions about how to avoid pain and exhaustion could be experienced as a limitation of meaningful activities. However, by accepting and adhering to the instructions their pain decreased.

“*Then she (the physiotherapist) asked me, why are you running? I guess you run uphill as well? Stop doing that because it will harm you. You could either swim or ride a bike. So, I quit jogging immediately and got rid of the pain.*”

(P7)

Being in a state of comprehensibility also meant actively paying attention to one’s body through ***self-monitoring*** and ***evaluating*** various trial and error experiences. The participants kept track of the number of activities and number of steps during a day to find the optimum level of physical activity without becoming exhausted or having pain.

“*I usually keep track of my walking, how many steps I take. Usually, I take between three and five thousand steps every day. That is what I manage before I get very tired.*”

(P10)

A barrier to comprehensibility was uncertainty. In addition to the uncertainty about whether symptoms were due to LEoP or old age, many expressed uncertainties regarding where to turn for assistance for their LEoP. They sensed a general lack of knowledge among healthcare staff and had to approach different units instead of one that was specialized in the rehabilitation of LEoP. There were also doubts pertaining to how to be sufficiently physically active as they were uncertain about the right balance between being active and at the same time not risking overload. As one participant put it:

“*First, I heard that I shouldn´t exercise because it would harm my muscles. Then, I was told I should exercise to some extent. But what level should it be at? I would really like to know a bit more about that.*”

(P13)

### 3.2. The Meaning of Manageability

Manageability was the interplay between the state of motion and the state of being. The state of motion in manageability was a process towards greater manageability, independence and autonomy. The state of being was the acceptance of dependency and the management of daily life. Both states involved active choices and the ability to switch between the states enhanced manageability. The change between the state of motion and state of being had a purpose, as accepting support enabled the person with LEoP to manage better and thereby experience independence and autonomy despite the need for assistance.

The motion towards greater manageability meant ***initiating interventions and clarifying needs***. The participants described many initiatives, such as self-referral to rehabilitation. Some services were paid for, e.g., ordering food for a dinner party instead of cooking themselves, which saved their energy for social interactions. There were also clever interventions, e.g., the development of a specialized wheelbarrow for the garden. ***Modifying the home environment*** was a significant part of their routines. Many had adapted their houses to facilitate various activities, e.g., installed a stair lift, toilet seats that could be adjusted to different heights, bathtubs made for sitting and adjustable tables and chairs.

“*Climbing stairs is really difficult. And we have made it easier by simply installing a stair lift and I climb the stairs as little as possible, that’s not something one really has to do.*”

(P2)

***Making plans*** was also important and involved different precautions, such as planning activities according to their abilities and thinking about their future when re-designing their houses or gardens.

“*We built an outdoor room a few years ago, and I stood up for myself that there mustn´t be any stairs, it must be on one level because I must be able to walk there with a rollator… So, we think ahead, my husband and I, when we do things, so that I can manage them later on. Yes, its´s always present, facilitating for the future…*”

(P11)

The state of being meant accepting dependency by ***receiving instrumental and social support***, such as assistance with everyday life chores and using various aids or devices. The various forms of instrumental support from society involved many different aids, e.g., orthopedic shoes, orthoses, crutches, disability-friendly alterations in the house, scooters, transportation services and home care services, enabling everyday life to function well.

“*I got an electric scooter. Pure joy! It was the best thing that has happened to me. I am free now. My husband rides his bike and he and our grandchildren walk beside me when I use the scooter. Like the easiest thing in the world. It’s great!*”

(P11)

Social support, such as daily help with things they found demanding, was mainly provided by spouses, relatives and neighbors. This was most valued and seemed to be very important for the sense of living a good life.

“*They (the grandchildren) help and support me when I need help and see that something needs to be carried in or out, for example. There is immediately someone who takes care of it. So, I get assistance with things they know I find difficult.*”

(P2)

The state of being also involved managing daily life with a disability. ***Keeping oneself occupied*** meant that some hobbies and activities were undertaken to occupy themselves at times when they had nothing else to do. For some, quiet activities had replaced those that could not be managed anymore due to disability.

“*After reading the newspaper I park myself at the computer, only taking a break for lunch and coffee. And basically, what I do is genealogy.*”

(P1)

***Adapting and achieving balance*** was important for ensuring that the strains of everyday life did not become too high.

“*I have let go of the thought that I must be so good and do everything and have everything ready and so on. I can now think that, no, I have already done that, it’s enough.*”

(P4)

This also meant that they had to do things at their own speed, often with breaks during the activity.

“*Mowing the lawn is quite difficult, but I manage anyways. I take breaks in between*”

(P8)

A barrier to manageability seemed to be a lack of participation and not being allowed to define one’s own needs. The participants did not always receive the support from society that they needed and unnecessary bureaucracy, rigid laws and regulations were sometimes experienced.

“*I wish there were less bureaucracy…. When I wanted to visit my daughter, I suggested that I could drive to (her hometown) and the stair climber could meet me there. But that was impossible…. I had to go from (participant’s home town) with the transportation service…. And I mean, it would have been a saving for everyone if I could use my own car.*”

(P9)

Furthermore, frustration and resignation were expressed when healthcare staff decided that one was not in need of treatment or assistance or determined the type of aid/assistance required, which had a negative impact on manageability.

### 3.3. The Meaning of Meaningfulness

The motion towards more meaningfulness meant nurturing aspects that the participants found interesting and meaningful. This involved the actions of ***using one’s inner drive for going forward*** *and **doing good and making a difference for others.*** In general, the participants seemed to have an inner drive that helped them to go forward towards their goals. This drive made them focus on possibilities instead of difficulties, confident that solutions could be found and willing to take on challenges.

“*We have been used to drive through Europe quite a lot, but one starts to get older, so that with car… but there are other travel choices…. Train, for example, I like traveling on a train.*”

(P5)

Doing good and making a difference for others was valuable for the participants, e.g., helping their spouse, letting others use their garden or doing voluntary work.

“*We are helping out by crocheting tiny dolls or animals that we give to the children’s hospital or to the ambulance service. They are for the children. I have made a lot of them.*”

(P2)

The motion towards meaningfulness also included the active choice of ***viewing things positively and valuing what one has by being mindful***. To view things positively was, e.g., to choose not to spend too much time on things that had gone wrong.

“*I don´t bury myself very easily for different things and I´m not a brooder either…. So that´s why I´m doing well, I guess that is helping me.*”

(P5)

Being mindful, i.e., living in the moment, was another active choice.

“*You know, it´s so wonderful just to sit down outside and just watch…. And with some coffee and so on. It’s not so bad. I can´t say more than that, but I mean… I enjoy and I value it.*”

(P6)

The state of being in meaningfulness involved ***enjoying nature*** as it made them happy and mindful. In particular, having a garden or summer house was much appreciated.

“*Where I live, I have a great view, which promotes mental wellbeing. High trees. Look how beautiful they are, close to the water and so on. I am lucky.*”

(P7)

***Enjoying meaningful social relationships*** meant being part of a social context, which resulted in a sense of comfort. The participants appreciated various interactive activities, e.g., book circles, excursions, the table tennis club and sewing circles. The COVID-19 pandemic had acted as a barrier and they were looking forward to resuming their social activities. ***Accessing financial security*** meant freedom to choose a preferred lifestyle and to add a bit of luxury to their ordinary everyday life.

“*We live a good life. Financially strong. It means a lot in our situation. We can allow ourselves certain things*”.

(P4)

***Being together with loved ones***, e.g., spouses, children, grandchildren and close friends, was very important for many participants. It prevented isolation and loneliness by providing a secure social belonging.

“*My life is good. I have my husband, children and grandchildren. Even great-grandchildren. We all keep in touch*”.

(P14)

Many participants expressed that a decline in their health would threaten meaningful activities, e.g., going for a walk.

“*I enjoy going for a walk in nature so much. I really hope I can continue with that for as long as possible*”.

(P12)

Being able to participate in social events was very important, and they hoped for a stable physical condition in the future.

## 4. Discussion

To the best of our knowledge, this is the first study that has explored the meaning of SOC among persons with LEoP in-depth. The novelty primarily lies in the profound understanding of SOC as both motion and being. The findings will be essential for rehabilitation professionals when providing self-management support to persons living with LEoP. This understanding also enriches the approach towards other persons with life-long conditions.

During the analysis, it became clear that the state of motion and the state of being were closely intertwined. In most sub-categories, there were traces of the other overarching theme, even if they mainly belonged to either motion or being. Some of the sub-categories were also of relevance for more than one category of SOC. These findings support Antonovsky’s statement that the categories of SOC are highly integrated [6]. Because of the novelty of SOC as a state of motion and being, we have chosen to discuss the findings based on the two overarching themes.

### 4.1. The State of Motion

The state of motion was characterized by active approaches, choices and actions. One active approach to achieve higher comprehensibility was to simplify information from rehabilitation professionals and make things concrete and understandable related to the consequences of LEoP. Contributory factors, such as old age and comorbidities, were frequently considered in the participants’ explanatory models of their current condition. However, the exact mechanisms behind LEoP, or if their current condition was due to LEoP or old age, did not appear to be important for the majority of the participants. This has previously been described by Sjödahl Hammarlund et al. [14] and can be seen as a coping behavior aimed at achieving a sense of normality. By “blaming old age” for the current condition, you are not very different compared to other persons of the same age [14]. Another active approach that our participants described was to continuously re-evaluate and identify patterns (i.e., understand cause and effect), possibly because their condition had changed from the time when they had their polio until now when they lived with LEoP. This gave them a sense of comprehensibility and a personal model of explanation for their current situation. Doing good and making a difference for others was an active approach that increased the participants’ sense of meaning. This approach seems to be important for meaningfulness, as it provides social meaning and a higher purpose. The same has also been described in traffic victims [15], in whom altruism was a coping behavior that increased a person’s meaning in life. Furthermore, in a study of older persons with a high level of SOC [16], voluntary work and helping others were very much appreciated by the participants, as they sensed it gave so much value in return.

Moreover, our participants chose to view things positively and to value what they had in life. This was important for meaning making, as it enabled a focus on values that were positive for them. Our findings are also in line with another study [16], where older persons with a high SOC were described as being satisfied, positive and forward looking.

The action of taking their own initiatives to increase manageability was often used by our participants, who made great efforts and clever interventions to improve their functioning. For example, some had designed their house or garden in a disability-friendly way and modified their home environment by using their own solutions or inventions. Similar efforts have previously been described among persons with LEoP [17]. Since childhood, they have been accustomed to struggling to overcome difficulties, making efforts to function in society and not differ from others [18]. Although some participants now received instrumental support from society it was sometimes not enough. Therefore, they had to take their own measures in their homes. Making plans was also important, both thinking ahead and planning how to perform challenging everyday activities. This was intended to increase manageability, both in the present and in the future. Our findings are also in line with another study of persons with LEoP, where the participants described planning every move in challenging situations in order to manage better [14].

The findings in the state of motion comprised aspects that could be related to using one’s inner drive for going forward. All the active approaches, choices and actions undertaken allowed the participants to go forward and focus on possibilities and aspects that gave them value in life. This is important for all categories of SOC and seems to be a fertilizer of the meaning making process, as it ultimately concerns the freedom to choose what one wants to do and to be able to do it. Similar findings have been described in a study of liver transplant recipients, where the freedom and ability to do what you want to do was found to be the meaning of health and quality of life [19].

### 4.2. The State of Being

The state of being included understanding and managing the demands of everyday life, as well as enjoying valued experiences and interactions. Listening to rehabilitation professionals’ advice and following their instructions were considered important for knowing how best to live with LEoP and engage in the right amount of daily activity. However, many participants expressed frustration about not knowing where to turn for help regarding their LEoP and frustration about being unaware of the right level of activity. Therefore, continuous self-monitoring and evaluation were important, e.g., to assess the number of steps/day as part of the daily routine in order to avoid physical overload. Similar findings were reported in a study of physical activity in persons with LEoP [20]. In that study, the participants needed to find a balance and adjust their activities in order to avoid fatigue and pain. To obtain individualized support from rehabilitation professionals, our participants wished for an “open door” to a rehabilitation clinic specialized in LEoP. This need has also been described by persons with muscular dystrophy [21] and is most likely applicable to all life-long progressive conditions. Even if the persons have lived with a disability for most of their lives, the progressiveness, albeit slow, means that new issues may arise, for which they might need support.

Accepting dependency by receiving instrumental and social support, keeping oneself occupied as well as adapting and achieving balance made it easier to manage the demands of everyday life. The importance of accepting one’s situation has also been described in persons with Parkinson’s disease, where such acceptance was necessary for their adaptation and to achieve life satisfaction [22]. In the present study, the acceptance of dependency interplayed with the state of motion (i.e., choosing independency). This gave the participants higher manageability, as acceptance enabled them to choose appropriate leisure activities and obtain the assistance they needed, thereby leading to more independence and autonomy. Instrumental support was to a great extent provided by society, while support from spouses, family and close friends was both instrumental and social. However, a barrier to manageability was the bureaucracy that sometimes made it impossible to obtain the instrumental support they needed. Similar experiences of a struggle with regulations and laws have been described among persons with muscular dystrophy [21]. Although the Swedish society welfare model is well developed and aims to provide every person with the support he or she requires, it sometimes appears to lack flexibility and the ability to meet individual wishes and needs.

The support from family and friends was greatly valued, as it gave the participants a sense of social belonging, of being cared for and the feeling of a good life. Having a good life also meant keeping oneself occupied and having meaningful everyday activities. The value of performing meaningful activities has also been described before [21,23,24,25] and is closely related to life satisfaction [26,27], highlighting the importance of minimizing barriers to participation. Adapting and achieving balance in everyday activities was part of their daily routine. This indicates that the participants were well adapted to their LEoP and no longer used their childhood coping behaviors, such as overachieving to blend in and not listening to the body [17].

Meaningfulness in everyday life was a source of energy for the participants and was found in nature, financial security and meaningful social relationships. Nature evoked joy and mindfulness, while financial security allowed them to live the life they wanted to live. Financial security makes things easier and as such, is a general resistance resource [6]. Enjoying meaningful social interactions gave a sense of belonging and being part of a context. Social contacts have previously been found important for SOC [16]. Having a social network can also be a general resistance resource, as it provides the person with the possibility of using the network as a resource for assistance [28]. In addition, being with loved ones was described as a source of meaningfulness in everyday life by our participants. As well as a sense of belonging, the closest relations seemed to give a feeling of being loved and accepted as one is. It is well established in the literature that regardless of the medical condition, social support and social belonging are essential aspects of life satisfaction, adaptation and experiencing health despite a disability [27,29,30,31,32]. Thus, we advocate that a strong focus on social belonging should be adopted among rehabilitation professionals and other healthcare professionals when managing people with a life-long disability.

### 4.3. Clinical Implications

The clinical implications of our findings are that the state of motion should be supported in order to increase comprehensibility, manageability and meaningfulness, and that barriers in the state of being should be reduced. To support the state of motion, rehabilitation professionals should be person-centered, listen to the person’s narrative and lived experiences, and form a partnership where self-management support can be provided. By understanding the person’s aspirations and meaning making, rehabilitation professionals could provide targeted and person-centered support. Persons’ own initiatives, such as modifying the home environment, and meaningful activities such as voluntary work should also be encouraged and supported.

To prevent barriers in the sense of being, the instrumental support that persons require should be offered and unnecessary bureaucracy avoided. It is also important to determine whether the person is in acceptance of his or her condition, as this facilitates adaptation. If not, Acceptance and Commitment Therapy (ACT) could be a useful strategy, which has been previously used in persons with lifelong conditions [33]. It is also important to determine if the person is still relying on coping behaviors learned during childhood, due to the fact that some of these behaviors, e.g., overachieving, are no longer adaptive. In such cases, ways to adapt and achieve balance should be taught. Different group activities, such as forums for persons with LEoP, should be offered in order to increase social belonging for persons who lack a social network.

One way to support the sense of motion and prevent barriers in the sense of being is through an interdisciplinary person-centered, goal-oriented rehabilitation program for persons with LEoP [3]. In such a program, participants are active team members in their own rehabilitation process and take responsibility for their own rehabilitation plan. In this plan, they describe their difficulties, needs and what they want to be able to do now and in the future. The increased insight into their abilities, limitations and goals could be a booster to their inner drive, starting a process of positive change through self-management. As LEoP is a progressive condition, albeit slow, they need the certainty of having somewhere to turn if they require assistance or rehabilitation later in life. Thus, lifelong follow-ups in a specialized rehabilitation unit would be highly beneficial.

Knowledge from this study could also be applied in the management and rehabilitation of other life-long progressive disabilities. LEoP share many similarities with long-term post-infectious pandemics such as COVID-19. It has been suggested that knowledge of SOC can be used in the interventions aimed at reducing the pandemic’s detrimental effects and promoting resilience [34]. In addition, we believe that knowledge of SOC could be applied in the management of frailty in old age [35].

### 4.4. Methodological Considerations, Strengths and Limitations

This study has several strengths. Men and women were equally represented and the age range of 65–84 years covers most persons with LEoP, at least in the western world. Their disability level also varied; some persons were quite ambulatory, whereas others used a wheelchair as their main mode of transportation. Both married/cohabiting and single persons were included, and those with different educational backgrounds, providing a rich source of experiences and thereby valuable insight into the meaning of SOC. The authors have different professional backgrounds (physicians specializing in rehabilitation medicine with extensive experience of persons with LEoP, a nurse, a physiotherapist and a neuropsychologist) and are members of rehabilitation teams with experience of different life-long conditions, studies of SOC and extensive experience of qualitative research. Thus, the authors’ pre-understanding of persons with LEoP was considered a strength in the interpretation of the results. Finally, the person who conducted the interviews (MN) had no previous relationship with the participants.

There are also some limitations. The participants were chosen from a cohort that had, at some point, taken part in an interdisciplinary rehabilitation program. Consequently, they had been provided with support from rehabilitation professionals and might thereby be more adapted to their condition than persons who have not taken part in such a program. In addition, all participants were of Swedish origin and all had an educational level higher than primary school. Therefore, transferability to persons who have not taken part in rehabilitation, persons of other origins and with lower education levels, as well as younger persons with LEoP is limited. As SOC is also related to cultural, political, and economic factors, further research on SOC in other populations and different national contexts is needed.

## 5. Conclusions

SOC in persons with LEoP existed in two overarching themes that were closely intertwined: a state of motion and a state of being. A profound understanding of SOC as both a state of motion and a state of being is essential for rehabilitation professionals when providing self-management support to persons living with LEoP, in order to increase their sense of living a good life. This understanding can also be used in the rehabilitation of other life-long conditions.

## Figures and Tables

**Table 1:** Characteristics of the 14 participants with Late Effects of Polio.

Gender	
Men, n (%)	7 (50)
Women, n (%)	7 (50)
Age (years)	
Mean (SD, range)	73 (5, 65–84)
Marital status	
Single, n (%)	2 (14)
Married/cohabitating, n (%)	12 (86)
Level of education	
Primary school (≤9 years), n (%)	0 (0)
Secondary school (10–12 years), n (%)	8 (57)
Higher education (≥13 years), n (%)	6 (43)

**Table 2:** An overview of the meaning of Sense of Coherence in persons with Late Effects of Polio.

**Sense of** **Coherence**	The meaning of Comprehensibility	State of motion	Simplifying by using metaphors and making things concrete	Re-evaluating information and identifying patterns		Developing personal models of explanation of illness
		State of being	Following instructions	Self-monitoring		Evaluating
	The meaning of Manageability	State of motion	Initiating interventions and clarifying needs	Modifying the home environment		Making plans
		State of being	Receiving instrumental and social support	Keeping oneself occupied		Adapting and achieving balance
	The meaning of Meaningfulness	State of motion	Using one’s inner drive for going forward	Doing good and making a difference for others		Viewing things positively and valuing what one has by being mindful
		State of being	Enjoying nature	Enjoying meaningful social relationships	Accessing financial security	Being together with loved ones

## Data Availability

All data were archived according to the Swedish Act concerning the Ethical Review of Research Involving Humans and are available upon reasonable request.
